# Supplementary material for: Lymphotoxin β Receptor: a Crucial Role in Innate and Adaptive Immune Responses against Toxoplasma gondii
Source: Infect Immun. 2021 May 17;89(6):e00026-21. doi: 10.1128/IAI.00026-21 (PMC8316152; doi:10.1128/IAI.00026-21)
Supplement: Supplementary file 1 [file iai.00026-21-s0001.pdf]

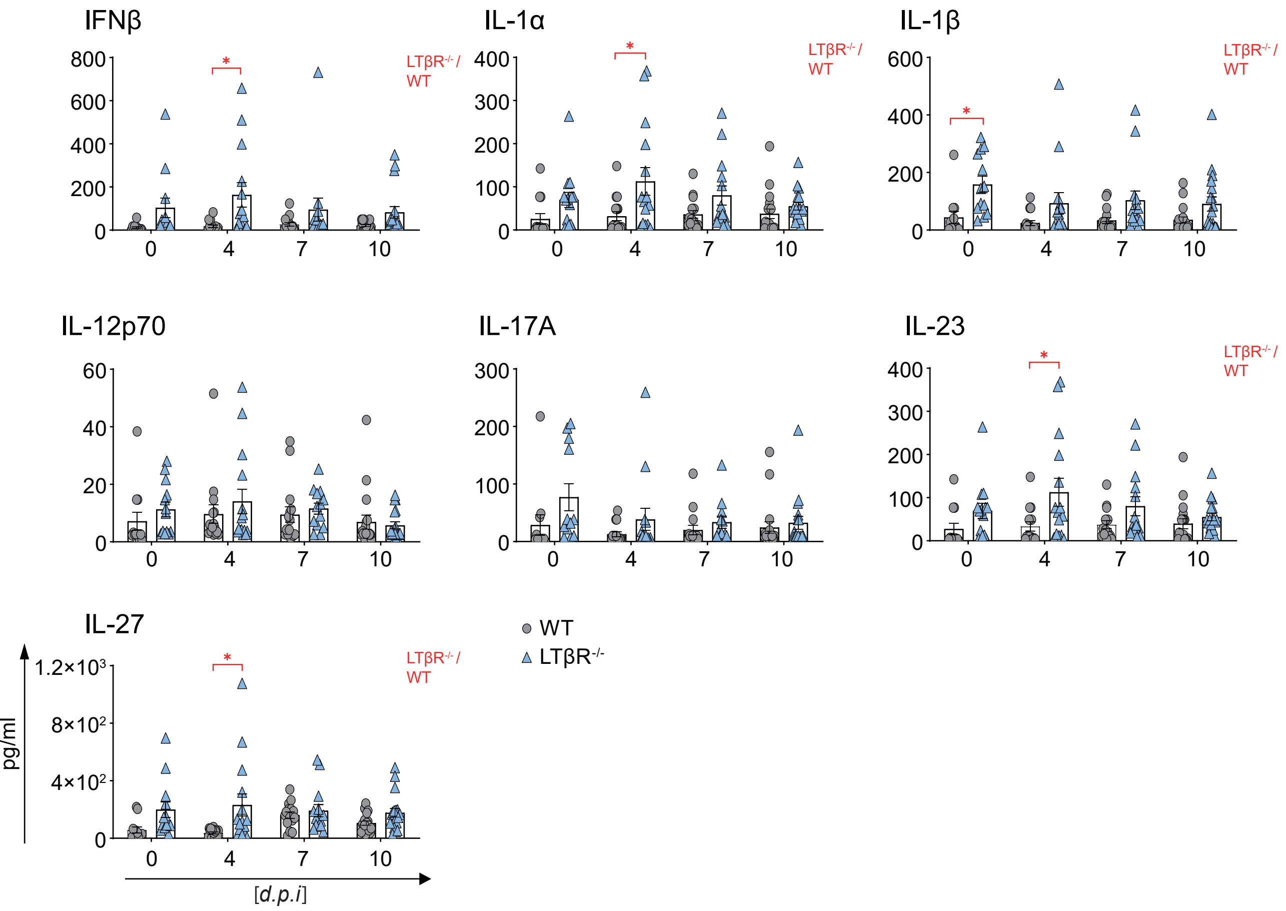

Suppl. Fig. 1│LTβR$^{-/-}$ mice show dysregulated cytokine expression. Expression of IFNβ, IL-1α, IL-1β, IL-12p70, IL-17A, IL-23 and IL-27 in the serum of uninfected and *T. gondii* infected WT and LTβR$^{-/-}$ mice (d0 - 7: n≥12, d10: n=18) analyzed via bead-based immunoassay. Data shown represent at least three independent experiments; symbols represent individual animals, columns represent mean values and error bars represent ± SEM. 2way ANOVA corrected for multiple comparison by the Tukey's post hoc test was used for statistical analysis. *P<0.0332.

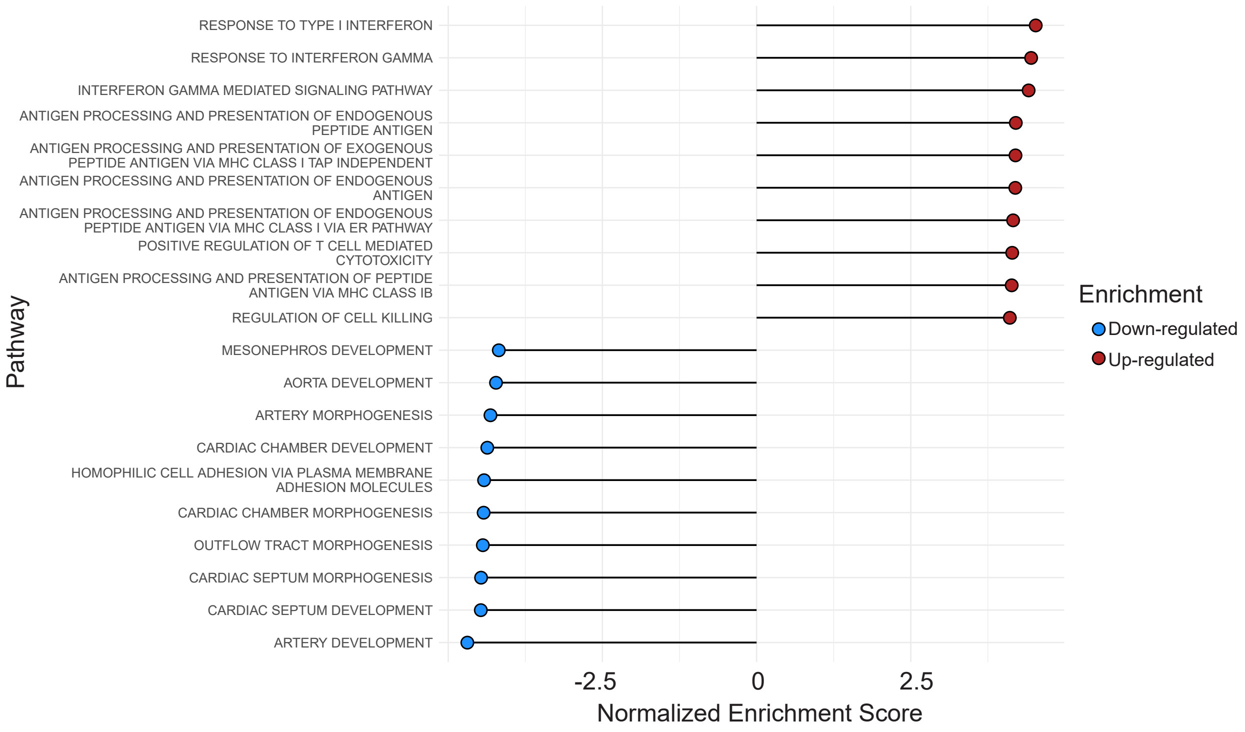

Suppl. Fig. 2│GSEA. GSEA generated from RNAseq data from lung tissue of *T. gondii* infected (ME49, 40 cysts; d7 *p.i.*) WT mice correlated to infected LTβR$^{-/-}$ mice (n=3/group). GO (Biological Process) molecular signatures were obtained using the msigdr package in R. Gene-level differential expression statistics from infected (d7 *p.i.*) WT mice were tested for geneset enrichment using the fgsea package, with an adjusted p-value of 0.1.

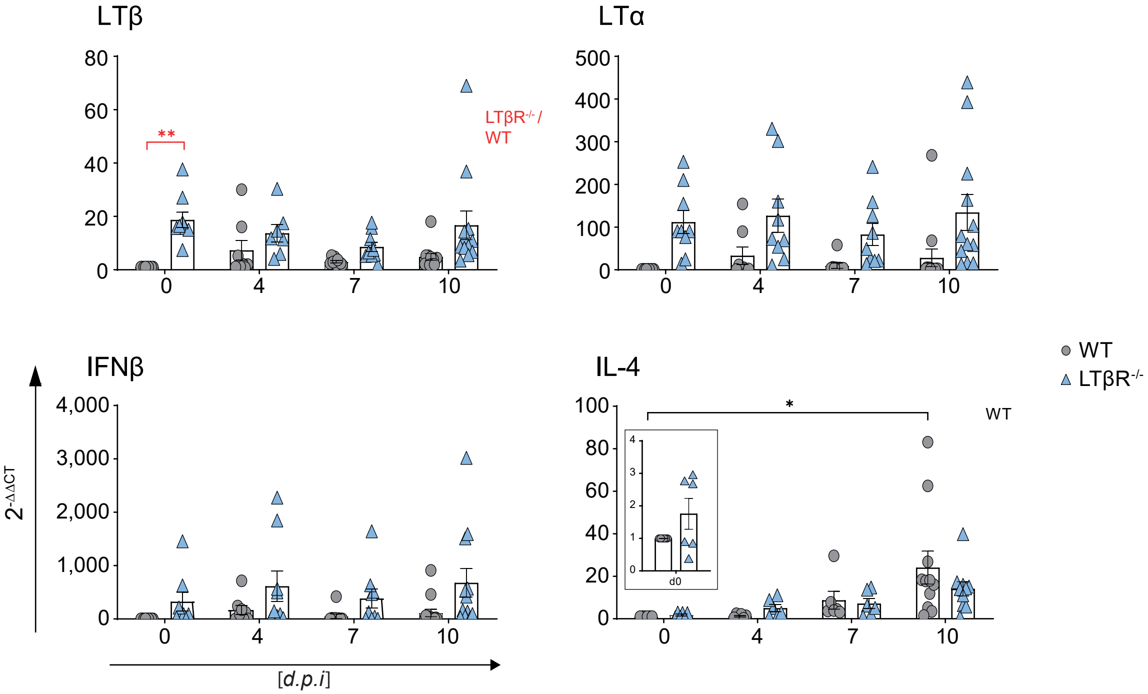

Suppl. Fig. 3|LTβR$^{-/-}$ mice show dysregulated cytokine expression in lung tissue. qRT-PCR of cytokines (LTβ, LTα, IFNβ, IL-4) in lung tissue from uninfected (d0) and *T. gondii* infected (ME49, 40 cysts *i.p.*) WT and LTβR$^{-/-}$ mice (d0 - 7: n≥12, d10: n≥14; exception: IL-4 n≥6, d0 – 10 *p.i.*). Data shown represent four independent experiments; symbols represent individual animals, columns represent mean values and error bars represent ± SEM. 2way ANOVA corrected for multiple comparison by the Tukey's post hoc test was used for statistical analysis. **P<0.0021.

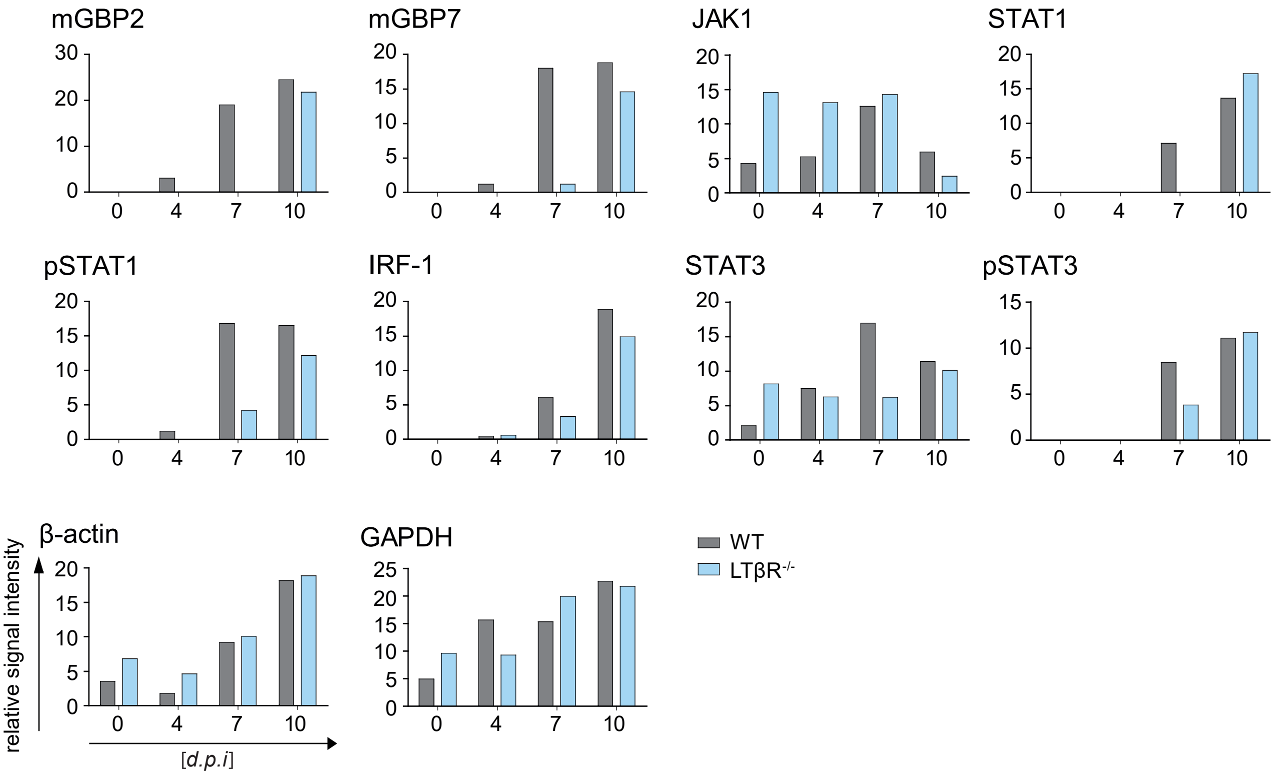

Suppl. Fig. 4│Immunoblot Quantification. Quantification of relative signal intensities of the immunoblot protein bands shown in Fig. 3c using ImageJ. Data shown are representatives of three independent experiments.

a

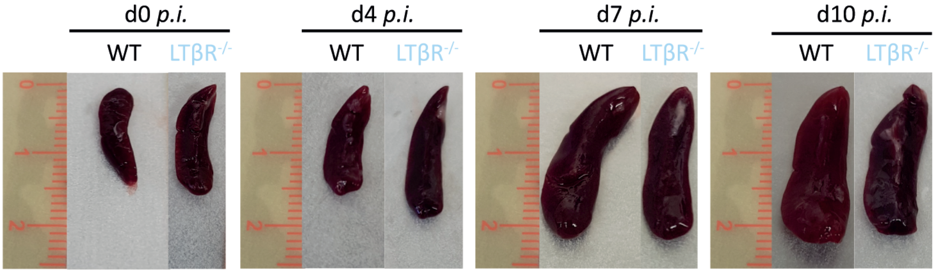

[*d.p.i.*]	WT [cm]	LTβR$^{-/-}$ [cm]
0	1.4	1.6
4	1.6	1.9
7	2.4	2.2
10	2.3	2.1

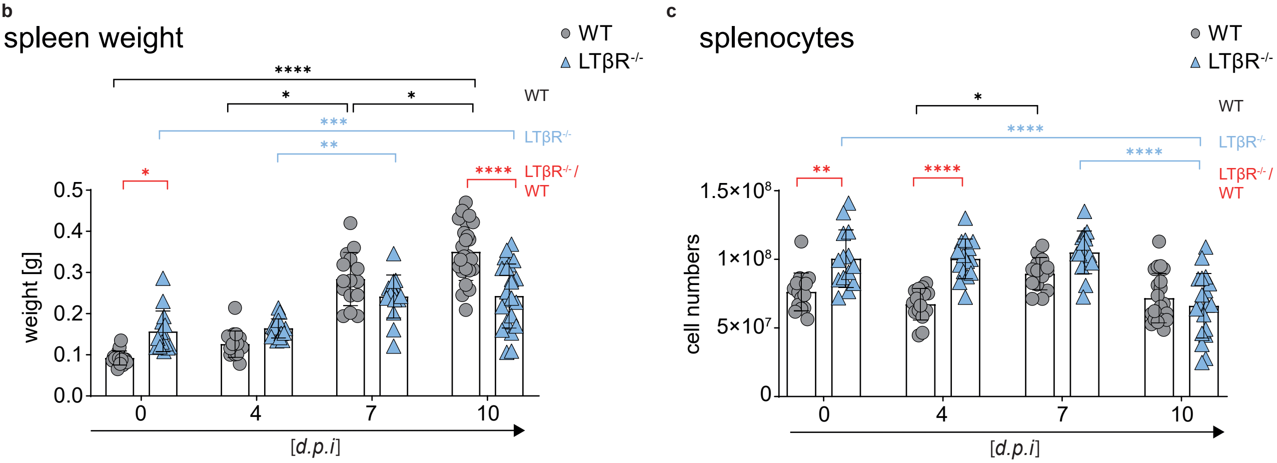

Suppl. Fig. 5 | Decreased spleen weight but increased splenocyte numbers. a, Depiction of representative spleens from uninfected (d0) and *T. gondii* infected (ME49, 40 cysts, *i.p.*) WT and LTβR$^{-/-}$ mice (d0 - 7 *p.i.*: n=9, d10 *p.i.*: n=≥9). Ruler indicated for scale [cm]. **b**, Spleen weight and **c** absolute splenocyte numbers from uninfected and *T. gondii* infected WT and LTβR$^{-/-}$ mice (d0 – 7 *p.i.*: n=15, d10 *p.i.*: n≥19). Data shown in **b** and **c** represent four independent experiments; symbols represent individual animals, columns represent mean values and error bars represent ± SEM. 2way ANOVA corrected for multiple comparison by the Tukey's post hoc test was used for statistical analysis represented in **b** and **c**. *P<0.0332, **P<0.0021, ***P<0.0002 and ****P<0.0001.

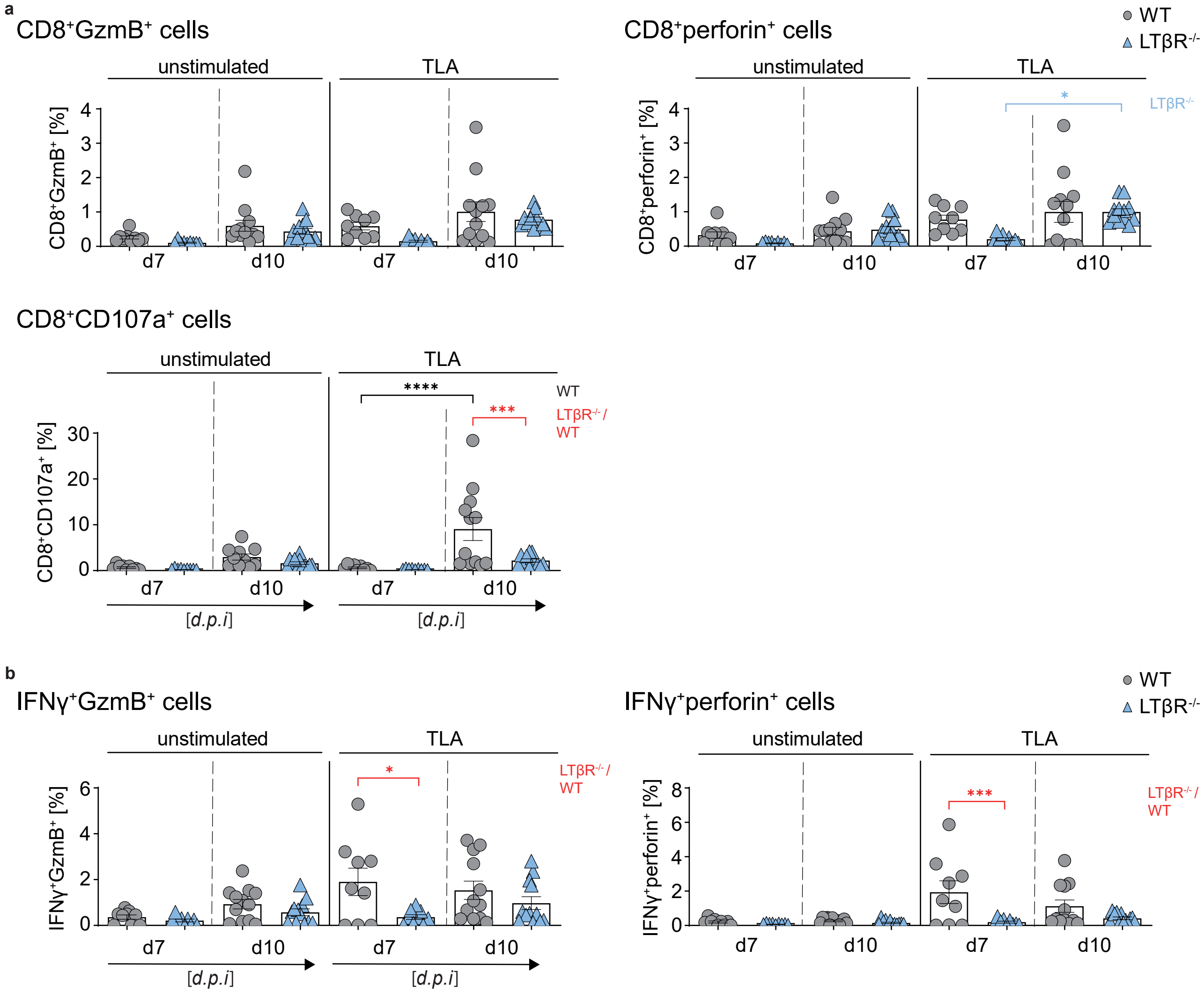

Suppl. Fig. 6 | LTβR deficiency impairs CD8 T cell effector function in the spleen. Intracellular staining of **a**, cytotoxic granule (GzmB⁺ or perforin⁺) containing and degranulating (CD107a⁺) CD8⁺ T cells and **b**, IFNγ⁺GzmB⁺ and IFNγ⁺perforin⁺ cells [%] of unstimulated and toxoplasma lysate antigen (TLA) *ex vivo* restimulated splenocytes from *T. gondii* infected (d7 and 10 *p.i.*) WT and LTβR$^{-/-}$ mice (d7: n≥6, d10: n≥10). Representative data of at least two independent experiments; symbols represent individual animals, columns represent mean values and error bars represent ± SEM. 2way ANOVA corrected for multiple comparison by the Tukey's post hoc test was used for statistical analysis. *P<0.0332, ***P<0.0002, ****P<0.0001.

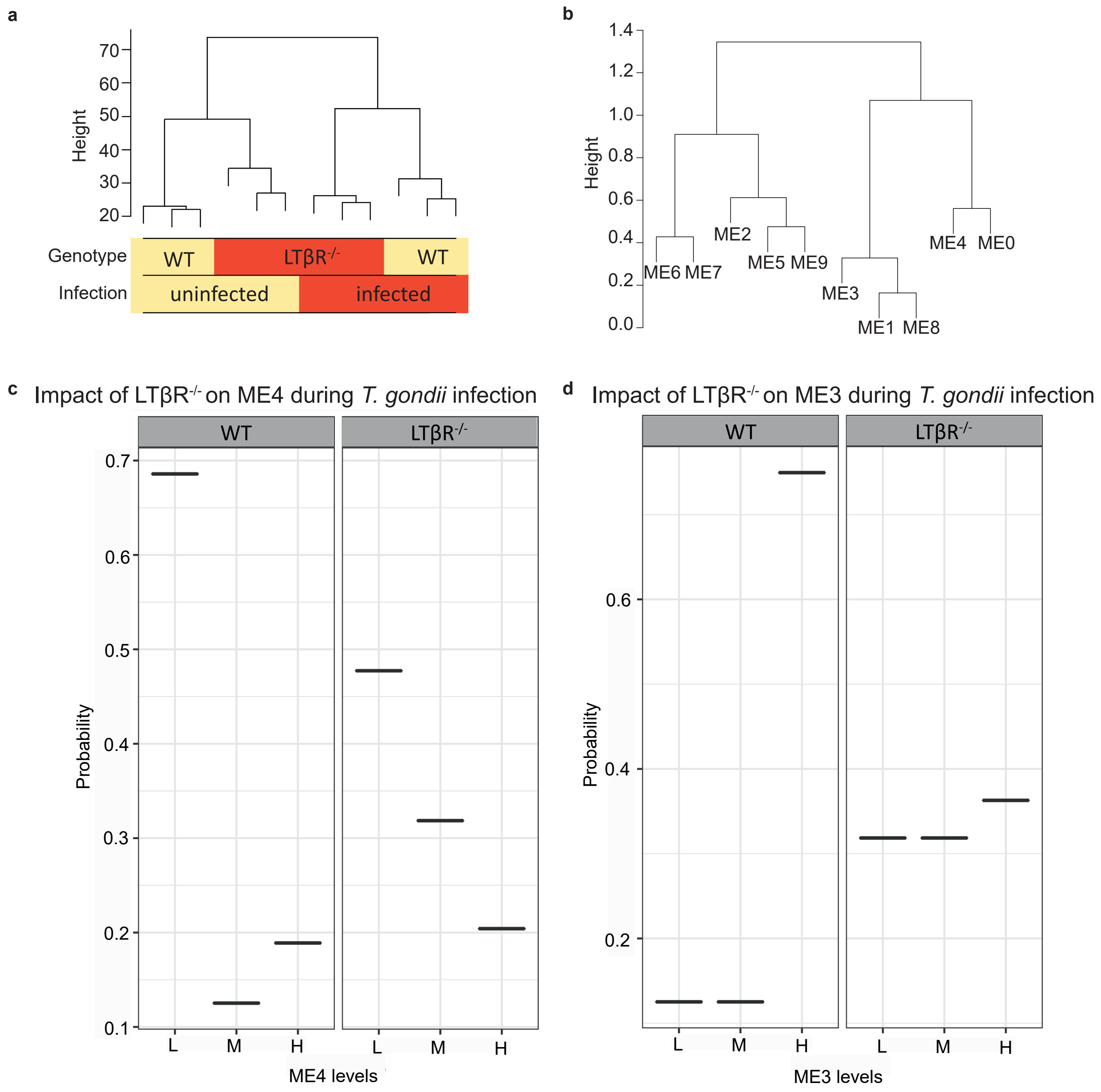

Suppl. Fig. 7│Samples, modules and bayesian interference of key relationships from RNAseq analysis. a, Sample dendrogram (hierarchical clustering) with trait heatmap of RNAseq data from lung tissue of uninfected (d0) and *T. gondii* infected (ME49, 40 cysts; d7 *p.i.*) WT and LTβR$^{-/-}$ mice (n=3). Expression data was filtered for differentially expressed genes (all conditions ~10,000 genes). Hierarchical clustering is illustrated as sample dendrogram including a trait heat map (red and yellow bars) for sample classification. For infection, the yellow bar shows clustering of uninfected (d0) and the red bar shows clustering of infected (d7 *p.i.*) animals. For genotype, yellow bars show clustering of WT and the red bar shows clustering of LTβR$^{-/-}$ animals. **b**, For module detection GmicR package in R was used, using a minimum module size of 30, mergeCutHeight of 0.3, and Rsquared cut of 0.80. Gene expression data was condensed into ten module eigengenes (ME0-9) before being used for generation of a host-pathogen network. **c**, The probability of ME4 levels during infection for WT and LTβR$^{-/-}$ mice is shown. ME4 shows a slight increase to the probability of being in a high expression level (represented as "H") in LTβR$^{-/-}$ mice. **d**, The probability of ME3 levels during infection for WT and LTβR$^{-/-}$ mice is shown. ME3 shows reduced probability of being in a high expression level (represented as "H") in LTβR$^{-/-}$ mice. Bayesian inference of the GmicR network was carried out using the bnlearn and gRain packages.

Supplementary Table 1: Primer and probes qRT-PCR.

**mGBP1**	**41**	**fwd**	**CAGACTCCTGGAAAGGGACTC**
		rev	CTTGGACCTGGAACATTCACTGAC
**mGBP2**	17	fwd	TGAGTACCTGGAACATTCACTGAC
		rev	AGTCGCGGCTCATTAAAGC
**mGBP3**	21	fwd	GGCTGAGGACTGTCCCTGT
		rev	CATGGTCCACTCGGAAGC
**mGBP5**	48	fwd	TCACTGAAGCTGAAGCAAGG
		rev	GCGTCAAAAACAAAGCATTTC
**mGBP6/10**	6TM*	fwd	ATATTTCAACATTTTTTGTTCCTTGT
		rev	GAAATGGGAGAAAAAATAAATGAAGC
**mGBP7**	93	fwd	GCAGAGAATCCGGTGCAG
		rev	TTTCCACTAGGCACACAGGA
**mGBP8**	8TM*	fwd	AAGAAGCTGAAGGAACAAAAGGC
		rev	GAAATGGGAGAAAAAATAAATGAAGC
**mGBP9**	9TM*	fwd	TTCCAAAACTTTCTCCAGTCACAGTA
		rev	GGCACGCTCCTCTGCAA
**IFNγ**	63	fwd	ATCTGGAGGAACTGGCAAAA
		rev	TTCAAGACTTCAAAGAGTCTGAGGTA
**IFNβ**	95	fwd	CAGGCAACCTTTAAGCATCAG
		rev	CCTTTGACCTTTCAAATGCAG
**iNOS**	13	fwd	CTTTGCCACGGACGAGAC
		rev	TGTACTCTGAGGGCTGACACA
**IL-4**	2	fwd	CATCGGCATTTTGAACGAG
		rev	CGAGCTCACTCTCTGTGGTG
**LTα**	62	fwd	TCCCTCAGAAGCACTTGACC
		rev	GAGTTCTGCTTGCTGGGGTA
**LTβ**	76	fwd	CCTGGTGACCCTGTTGTTG
		rev	TGCTCCTGAGCCAATGATCT
**TNFα**	49	fwd	TCTTCTCATTCCTGCTTGTGG
		rev	GGTCTGGGCCATAGAACTGA
**NOX2-pg91phox**	20	fwd	TGCCAACTTCCTCAGCTACA
		rev	GTGCACAGCAAAGTGATTGG
**IDO1**	2	fwd	GGGCTTCTTCCTCGTCTCTC
		rev	TGGATACAGTGGGGATTGCT
**β-actin**	106	fwd	TGACAGGATGCAGAAGGAGA
		rev	CGCTCAGGAGGAGCAATG
**TgB1**	TgB1*	fwd	GCTAAAGGCGTCATTGCTGTT
		rev	GGCGGAACCAACGGAAAT

Primer (Metabion, Martiensried,Germany); numbered probes (Universal ProbeLibrary, Roche, Mannheim, Germany);TM probes (TipMolBIOL, Berlin, Germany); TgB1 probe (Metabion, Martiensried,Germany):

6TM [5’ ->3’]: FAM-AGT CAT GTT CAA TCT TCT CCC TCT TGT CC-BHQ1
8TM [5’ ->3’]: FAM-TGT TTC AGT TGC TGT ATC TCT CCG TCC A-BHQ1
9TM [5’ ->3’]: FAM-CCA GCA GTG AGG GCT CTA TCT GCC T-TMR
TgB1 [5’->3’]: FAM-ATC GCA ACG GAG TTC TTC CCA GAC GT-BHQ1

Supplementary Table 2: Antibodies immunoblots.

Primary antibodies	Host species	Company
**anti-Jak1 (D1T6W)**	mouse	CST
**anti-Stat1**	rabbit	CST
**anti-p-Stat1 (Tyr701)**	rabbit	CST
**anti-IRF-1 (D5E4) XP®**	rabbit	CST
**anti-Stat3 (79D7)**	rabbit	CST
**anti-p-Stat3 (Tyr705) (D3A7) XP®**	rabbit	CST
**anti-mGBP2**	rabbit	Eurogentec
**anti-mGBP7**	rabbit	Eurogentec
**anti-β-Actin (8H10D10)**	mouse	CST
**anti-GAPDH)14C10)**	rabbit	CST
abcam, Cambridge, UK; Cell Signaling Technology (CST), Frankfurt, Germany; Eurogentec, Lüttich, Belgium		
